# Evidence‐based standardized sample handling protocol for accurate blood‐based Alzheimer's disease biomarker measurement: Results and consensus of the Global Biomarker Standardization Consortium

**DOI:** 10.1002/alz.70752

**Published:** 2025-09-30

**Authors:** Inge M. W. Verberk, Mariam Gouda, Daniel Antwi‐Berko, Mardou van Leeuwenstijn, Bram Bongers, Isabel M. Houtkamp, Wiesje M. van der Flier, Shorena Janelidze, Oskar Hansson, Nathalie Le Bastard, Manu Vandijck, Jacob Hunter, Lee Honigberg, Kristopher M. Kirmess, Philip B. Verghese, Kaj Blennow, Henrik Zetterberg, Emily A. Meyers, Rebecca M. Edelmayer, Charlotte Teunissen

**Affiliations:** ^1^ Neurochemistry Laboratory Laboratory Medicine Amsterdam UMC Vrije Universiteit Amsterdam Amsterdam Neuroscience Amsterdam the Netherlands; ^2^ Alzheimer Center Amsterdam Neurology Vrije Universiteit Amsterdam Amsterdam UMC location VUmc Amsterdam the Netherlands; ^3^ Department of Computing and Information Sciences Department of Biology AI Technology for Life Utrecht University Utrecht the Netherlands; ^4^ Amsterdam Neuroscience Neurodegeneration Amsterdam the Netherlands; ^5^ Epidemiology and Data Science Vrije Universiteit Amsterdam Amsterdam UMC location VUmc Amsterdam the Netherlands; ^6^ Department of Clinical Sciences Clinical Memory Research Unit Lund University Lund Sweden; ^7^ Fuijirebio Europe N.V. Ghent Belgium; ^8^ ALZpath Carlsbad California USA; ^9^ C2N Diagnostics LLC St Louis Missouri USA; ^10^ Institute of Neuroscience and Physiology Sahlgrenska Academy at the University of Gothenburg Gothenburg Sweden; ^11^ Clinical Neurochemistry Laboratory Sahlgrenska University Hospital Mölndal Sweden; ^12^ Department of Neurodegenerative Disease UCL Institute of Neurology Queen Square London UK; ^13^ UK Dementia Research Institute UCL London UK; ^14^ Hong Kong Center for Neurodegenerative Diseases InnoHK Hong Kong China; ^15^ Wisconsin Alzheimer's Disease Research Center University of Wisconsin School of Medicine and Public Health University of Wisconsin‐Madison Madison Wisconsin USA; ^16^ Centre for Brain Research Indian Institute of Science Bangalore India; ^17^ Alzheimer's Association Chicago Illinois USA

**Keywords:** amyloid beta, glial fibrillary acidic protein, neurofilament light, phosphorylated tau, plasma biomarkers, pre‐analytical variability, pre‐analytics, sample handling, stability

## Abstract

**INTRODUCTION:**

Blood‐based biomarkers (BBMs) have revolutionized Alzheimer's disease diagnosis and monitoring. Their pre‐analytical stability requires scrutiny. This study assessed pre‐analytical effects to inform a standardized sample handling protocol.

**METHODS:**

Assessed pre‐analytical variations included collection tube type, hemolysis, centrifugation settings, centrifugation/storage delays, tube transfers, and freeze‐thawing (*n* = 15/experiment). Phosphorylated tau (pTau) isoforms were measured with Simoa, Lumipulse, MesoScale Discovery, and immunoprecipitation‐mass spectrometry. Amyloid‐beta (Aβ42, Aβ40), glial fibrillary acidic protein (GFAP), and neurofilament light (NfL) protein were measured with Simoa.

**RESULTS:**

All assessed BBM levels varied by over 10% by collection tube type. Aβ peptides were the most sensitive, and their levels declined >by more than 10% under storage and centrifugation delays, more steeply at room temperature (RT) compared with 2°C to 8°C. NfL and GFAP levels increased by more than 10% upon RT/−20°C storage. pTau isoforms demonstrated stability across most pre‐analytical variations.

**DISCUSSION:**

We established an evidence‐based handling protocol to ensure reliable sample handling for neurological BBMs upon adoption in clinics, trials, and research.

**Highlights:**

- Sample handling protocols can mitigate pre‐analytical effects on BBM results.
- We developed an evidence‐based, expert‐consensus plasma sample handling protocol.
- Primary collection tube and delays to centrifuging or freezing impact AD BBMs.
- Plasma pTau217 is highly resistant to pre‐analytical sample handling variations.
- Plasma Aβ42 and Aβ40 were most sensitive to pre‐analytical variations.

## 1 BACKGROUND

Blood‐based biomarkers (BBMs) are revolutionizing Alzheimer's disease (AD) research and diagnostics.^1^, ^2^ They are minimally invasive, accessible, cost‐effective, sustainable, and potentially reliable alternatives to the current diagnostic biomarker methods: positron emission tomography (PET) scans and cerebrospinal fluid (CSF) biomarker analysis.^1^, ^3^ With the introduction of anti‐amyloid disease‐targeting treatments, there is an essential need to incorporate BBMs into clinical practice to meet the increased demand for AD biomarker assessment and monitoring.^4^, ^5^

The recently revised AD diagnosis and staging criteria of the Alzheimer's Association workgroup incorporated the following BBMs into their framework: core AD BBMs are amyloid beta1‐42/amyloid beta1‐40 (Aβ42/Aβ40) ratio, phosphorylated mid‐region tau (pTau) variants (pTau181, pTau217, pTau231), and pTau/Aβ42 ratio.^2^ These BBMs reflect amyloid and early tau proteinopathy.^2^ Non‐core BBMs neurofilament light (NfL) and glial fibrillary acidic protein (GFAP) reflect neurodegeneration and astrocyte reactivity, which are important pathogenic processes that play a role in AD,^2^ as well as in other neurological diseases (e.g., frontotemporal dementia, multiple sclerosis).^6^, ^7^ These core and non‐core BBMs may be used in clinical practice if the plasma assays achieve acceptable performance criteria.^4^, ^8^ Specifically, plasma pTau217 assays that meet acceptable accuracy standards, potentially in combination with other core or non‐core plasma biomarkers, could be used for the diagnosis of AD.^2^, ^9^ Numerous studies show that the diagnostic performance of plasma pTau217 might be comparable to that of the gold standard CSF AD biomarkers and amyloid PET.

The widespread clinical validation and adoption of the AD‐related BBM assays can be hindered by variability in pre‐analytical sample handling, as this can substantially affect BBM levels and consequently their diagnostic accuracy.^1^, ^10^, ^11^ There is considerable variability in sample handling across AD biobank studies.^11^ Through collaboration with the Alzheimer's Association, we therefore published an initial evidence‐based sample handling protocol to mitigate any pre‐analytical sample handling effect on BBM measurements, which at that time was primarily based on Aβ42 and Aβ40 measurement stability.^11^ Among the AD‐related BBMs previously assessed, Aβ42 and Aβ40 measurements appeared most susceptible to sample handling variations, particularly for centrifugation and −80°C storage delays.^11^, ^12^, ^13^, ^14^, ^15^, ^16^, ^17^ Specifically, it was found that Aβ42 and Aβ40 values decreased by more than 20% when centrifugation of whole blood or −80°C freezing of EDTA plasma was delayed by 24 h while tubes were kept at room temperature (RT).^11^, ^12^ NfL and GFAP appeared only modestly affected by variability in sample handling, as less than 10% change in levels was observed upon centrifugation and storage delays.^11^, ^12^, ^18^, ^19^ Some studies examined the influence of pre‐analytical sample handling effects on pTau217 and pTau181 measurements^11^, ^12^, ^14^, ^15^, ^18^, ^19^, ^20^ and suggested that measurements of pTau were quite resistant to sample handling variations. However, a cross‐technology and cross‐assay comparative pre‐analytical study comprehensively covering the pre‐analytical phase was still lacking, which is essential to disentangle technical analysis variation from protein measurement (in)stability.^8^

Supported by the Alzheimer's Association and led by the Global Biomarker Standardization Consortium (GBSC) and the Standardization of Alzheimer's Blood Biomarkers (SABB) workgroup, this study aimed to verify and refine our initially published guideline.^11^ Specifically, we systematically evaluated the impact of diverse pre‐analytical sample handling variations on key AD BBMs but now with a main focus on pTau, utilizing multiple analytical technologies and pTau assays. In addition, we included samples from both patients with AD and healthy controls (HCs) to cover the broad dynamic range of the BBM assays. Lastly, we extended certain pre‐analytical conditions to more extreme scenarios to capture realistic handling scenarios outside specialized centers in more remote settings. We here present the refined, comprehensive, evidence‐based, and consensus blood sample handling protocol, which mitigates the influence of pre‐analytics on key neurological BBM measurements and their interpretation.

## 2 METHODS

### 2.1 Samples and pre‐analytical experiments

At Amsterdam University Medical Centers (Amsterdam UMC), venous blood samples were obtained from 85 individuals who presented at the outpatient clinic for a blood draw for any disease as controls. In addition, 81 patients with AD‐positive CSF biomarkers or an abnormal amyloid PET scan were prospectively included from the Alzheimer Center Amsterdam. Blood samples obtained from individuals were distributed among 11 pre‐analytical experiments so that a total of 15 individuals per experiment were included, of whom eight were controls and seven were patients with AD. A sample size of *n* = 15 was previously determined based on paired two one‐sideded equivalence power calculation test, assuming 10% as a relevant change. Blood samples were processed anonymously, and no demographic or clinical information was collected.

**RESEARCH IN CONTEXT**

1. **Systematic review**: We reviewed PubMed literature on the impact of pre‐analytical variations on blood‐based biomarkers (BBMs) in Alzheimer's disease (AD) and neurodegeneration. Existing literature focused on a single analytical platform, investigated a narrow range of pre‐analytical variations, or did not include the most promising AD biomarker, plasma phosphorylated‐tau217. We evaluated the influence of a wide range of pre‐analytical variations across multiple platforms and biomarkers, to establish a technology‐independent, evidence‐based sample handling protocol for reliable BBMs measurement.
2. **Interpretation**: Our findings highlighted prominent pre‐analytical variations that require strict standardization, such as primary collection tube used, delays in centrifugation and delays in ‐80°C freezing. The most susceptible BBMs were Aβ42, Aβ40 and pTau217/Aβ42 ratio. pTau isoforms, GFAP and NfL were more resistant to sample handling variations.
3. **Future directions**: We recommend adoption of the developed pre‐analytical sample handling protocol in clinical and research settings.

**FIGURE 1:**
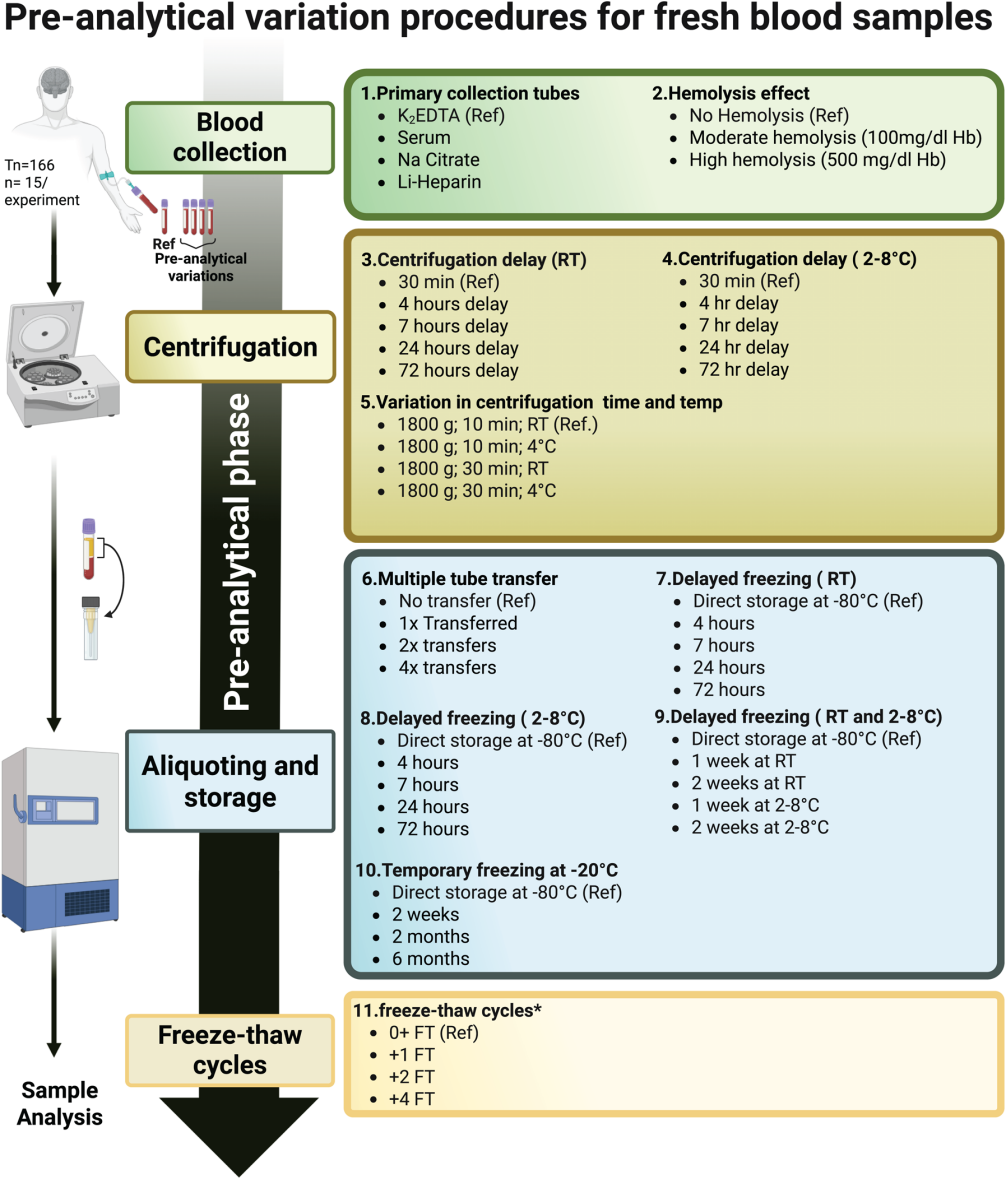
Overview of the 11 pre‐analytical experiments (*n* = 15/experiment) performed on fresh blood samples covering the complete blood processing workflow, including sample freeze‐thawing. Each pre‐analytical experiment has a reference condition and two to four experimental pre‐analytical variations. All experiments were performed on K_2_EDTA plasma samples, except experiment 1, which varied the primary blood collection tube. The hemolysis experiment was based on Delgado's method (strategy 1).^21^ Note, experiment 11, freeze‐thaw cycles, had only *n* = 5 for condition +4FT. FT, freeze‐thaw; Hb, hemoglobin; hr, hour; K_2_EDTA, dipotassium ethylenediaminetetraacetic acid; Li, lithium; Na, sodium; RT, room temperature.

Pre‐analytical experiments were designed systematically, where each pre‐analytical experiment included one reference condition and two to four different handling conditions compared to the reference. The reference condition was defined as a K_2_EDTA blood sample standing for 30 min at RT, followed by centrifugation for 10 min at 1800 x *g* at RT, after which, without any further delays, plasma was separated from whole blood, aliquoted into screw‐capped polypropylene storage tubes (here: 250 µL aliquots in 0.5 mL Sarstedt tubes), and stored at −80°C until use. All pre‐analytical experiments and conditions are summarized in Figure 1. The hemolysis experiment was based on Delgado's method (strategy 1).^21^ The maximum durations selected for each pre‐analytical condition were based chosen as a continuation of our earlier work^11^ and guided by experimental feasibility. The complete pre‐analytical sample set consisted of 720 samples divided over several aliquots per condition and covered the complete blood processing workflow: sampling, centrifugation, pooling, −80°C freezing, and sample freeze‐thawing.

This study was performed according to the Declaration of Helsinki and approved by the medical ethics committee of the Amsterdam UMC, location VUmc (approval number: 2019.257‐NL69123.029.19). All individuals agreed to participate in this study by signing an informed consent form to use their blood material for the purpose of the study.

### 2.2 Sample measurements

Aliquots of the pre‐analytical sample sets were distributed on dry ice among multiple centers, each utilizing a distinct technology and assay for the BBM measurements. Measurements were performed according to the manufacturer's protocols. Fujirebio measured pTau217 and pTau181 in singulate using the LUMIPULSE G1200 instrument (Fujirebio Europe N.V.; pTau181 was measured in one‐time freeze‐thawed samples). C2N Diagnostics measured pTau217 and non‐pTau217 (npTau217) using their standard operating procedure for the %pTau217 test, employing the immunoprecipitation liquid chromatography–mass spectrometry (LC‐MS) method, with a sample pre‐dilution step (750 µL sample, 250 µL diluent). %pTau217 was calculated by dividing pTau217 by npTau217 levels (%p‐tau217 = p‐tau217/np‐tau217 *100). Lund University measured pTau217 using the Eli Lilly assay in duplicate on the MesoScale Discovery (MSD) platform.^22^, ^23^ The Neurochemistry Laboratory, Amsterdam UMC, measured pTau217 in duplicate using the ALZpath assay on the Simoa HD‐X analyzer. In addition, to compare the results of this study to the published SABB‐GBSC sample handling procedure,^11^ we quantified pTau181 using the commercial pTau181 V2 assay in duplicates (Quanterix, Billerica, USA) and NfL, GFAP, Aβ42, and Aβ40 using the commercial Neurology 4‐plex E assay (Quanterix, Billerica, MA, USA) in singulate on the Simoa HD‐X analyzer. The pTau217/Aβ42 ratio was calculated by dividing the Lumipulse pTau217 levels by the Aβ42 Simoa levels.

### 2.3 Data analysis

Data from both the reference and experimental conditions were excluded if the measurement in the reference condition was below the limit of detection or had a coefficient of variation greater than 20% between duplicates. However, if such issues occurred only in the experimental conditions and not in the reference condition, the values were retained, as they may reflect true pre‐analytical effects rather than analytical variability. Levels of BBMs measured in experimental conditions were normalized to the levels measured in the reference condition as follows: Recovery (%) = ([experimental condition]/[reference condition])*100. If a > ±10% change in the median recovery was observed, then the pre‐analytical handling effect was considered relevant.^11^ In addition, the statistical significance of differences in experimental conditions compared to the reference condition was assessed using the Wilcoxon signed‐rank exact test. Data were analyzed and visualized using R version 4.3.2.

### 2.4 Establishment of SOP

When a pre‐analytical variation condition led to both a relevant change (> ±10% change in recovery) and a statistically significant change (*p* < 0.05), we deemed this condition to impact the plasma biomarker measurement accuracy. If multiple assays/technologies were used to measure a biomarker, the condition was considered impactful if a relevant and statistically significant change was observed in at least one assay/technology. The impacting conditions were used to define acceptable handling parameters in a draft sample handling procedure, which was subsequently presented to the SABB‐GBSC in an online consensus meeting for finalization. To build consensus, an initial draft of the sample handling protocol, along with supporting data, was shared with members of the GBSC via an online forum. Feedback was requested from both academic and industry representatives and discussed during a virtual workgroup meeting. Suggestions were incorporated into the final protocol when the suggestions were supported by empirical evidence as obtained within this study.

## 3 RESULTS

### 3.1 Effect of pre‐analytical variation on pTau levels

**FIGURE 2:**
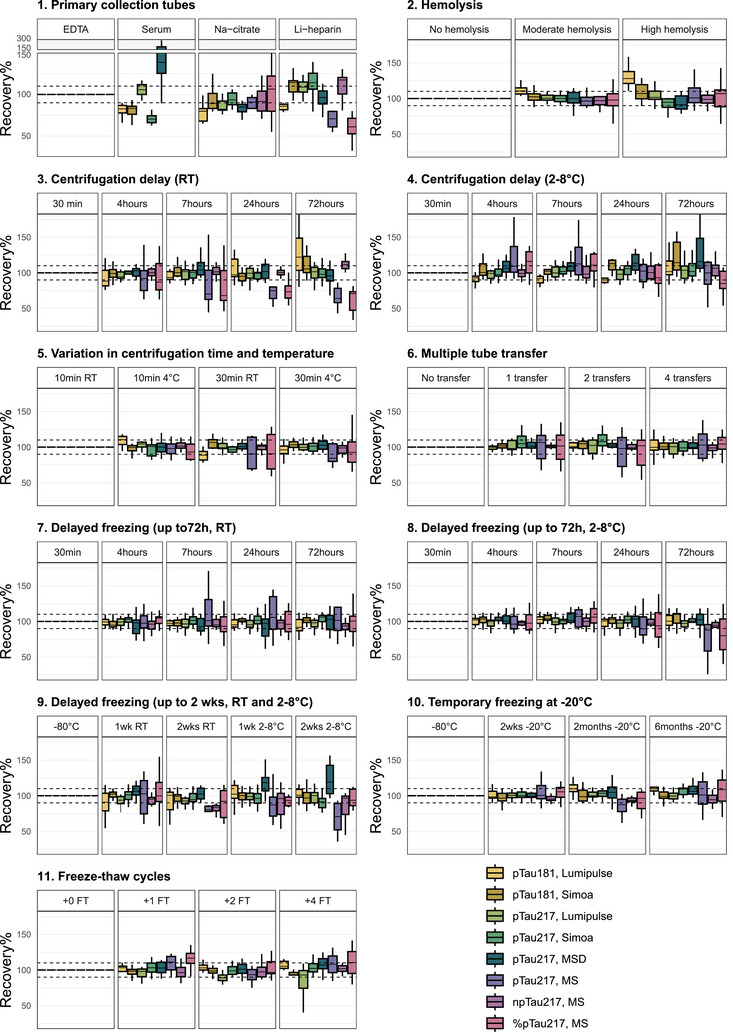
Recovery% plots visualizing the effect of 11 pre‐analytical variations on pTau181 and pTau217 levels as measured by Lumipulse, Simoa, MSD, and LC‐MS and npTau217 levels, as well as pTau217/npTau217 (%pTau217) ratio as measured by LC‐MS. Levels measured in the experimental condition were normalized to levels measured in the reference conditions, i.e., centrifugation of K_2_EDTA blood tubes at 1800 × *g* at RT after standing at RT for 30 min, followed by plasma separation and aliquoting in low binding polypropylene screw‐capped storage tubes and storage at −80°C without any delays. Horizontal dashed lines at 90% and 110% represent an allowable 10% change in experimental conditions compared to the reference condition. Box plots represent the median and interquartile range (IQR), whiskers represent the extreme data points that are within 1.5 times the IQR. LC‐MS serum measurements were below the limit of detection for most samples (data not shown). EDTA, ethylene diamine tetraacetic acid; FT, freeze‐thaw; LC‐MS, liquid chromatography‐mass spectrometry; Li‐heparin, lithium‐heparin; MSD, Meso Scale Discovery; Na‐citrate, sodium‐citrate; npTau, non‐phosphorylated tau; pTau, phosphorylated tau; RT, room temperature; Simoa, Single molecule array.

The effects of the 11 pre‐analytical experiments on levels of pTau181, pTau217, and npTau217 measured with Lumipulse, Simoa, MSD, and LC‐MS are presented in Figure 2 and Supplementary File 1 and are described in detail below.

### 3.2 Effect of primary collection tube

The type of primary collection tube used for blood withdrawal led to differences in measured levels of pTau181, pTau217, and npTau217 (Figure 2.1).

In serum, pTau181 was consistently measured at lower levels compared to K_2_EDTA plasma (both Lumipulse and Simoa: median recovery = −17%, *p* < 0.001). For pTau217, a more variable result was obtained. Levels of pTau217 were lower in serum compared to K_2_EDTA plasma when measured with Simoa (−30%, *p* < 0.001), higher when measured with MSD (+39%, *p* < 0.001), and similar when measured with Lumipulse. Note, with LC‐MS no reliable pTau217 and npTau217 results could be obtained in serum (respectively one and three out of 15 serum samples gave results; data not shown).

In Na‐citrate compared to K_2_EDTA plasma, pTau181 levels were lower when measured with Lumipulse (−20%, *p* = 0.001; not significant with Simoa: −11%, *p* = 0.277). pTau217 levels were lower when measured with Lumipulse (−18%, *p* < 0.001) and MSD (−15%, *p* < 0.001) but were similar in Na‐citrate and K_2_EDTA plasma when measured with Simoa and LC‐MS. In addition, LC‐MS npTau217 and %pTau217 were similar in level in Na‐citrate and K_2_EDTA plasma samples.

In Li‐heparin plasma compared to K_2_EDTA plasma, pTau181 levels were lower when measured with Lumipulse (−14%, *p* = 0.02) but higher when measured with Simoa (+14%, *p* = 0.003). pTau217 levels were similar in Li‐heparin and K_2_EDTA plasma when measured with Lumipulse, MSD, and LC‐MS, but higher when measured with Simoa (+14%, *p* = 0.021). LC‐MS npTau217 was higher (+17%, *p* = 0.02) and %pTau217 was lower (−39%, *p* = 0.03) in Li‐heparin compared to K_2_EDTA plasma.

### 3.3 Effect of hemolysis

Compared to no hemolysis, moderate hemolysis at 100 mg/dL Hb or high hemolysis at 500 mg/dL Hb (Figure 2.2) resulted in higher pTau181 levels when measured with Lumipulse (moderate: +11%, high: +28%; both *p* < 0.04) but not when measured with Simoa. Hemolysis did not alter the pTau217 levels as measured with any of the four technologies, nor did it alter the npTau217 level and %pTau217.

### 3.4 Effect of centrifugation delays

Compared to direct processing, when K_2_EDTA tubes were kept at RT prior to centrifugation (Figure 2.3), pTau181, pTau217, and npTau217 levels were stable up until the 7‐h timepoint, independent of the assay used. At the 24‐ and 72‐h timepoints, pTau217 levels were lower when measured with LC‐MS (−25%, *p* = 0.03; 72 h: −36%, *p* = 0.02), and pTau181 levels were higher when measured with Lumipulse (+22%, *p* = 0.01) at the 72‐h timepoint. pTau181 level measured with Simoa and pTau217 levels measured with Lumipulse, Simoa, and MSD were stable up until the 72‐h timepoint (i.e., the last timepoint assessed), as were LC‐MS npTau217 level and %pTau217.

When the K_2_EDTA tubes were kept in the fridge (2°C–8°C) prior to centrifugation, plasma pTau181, pTau217, and npTau217 levels were stable up until the 7‐h timepoint, independent of the assay used to measure the levels (Figure 2.4). At the 24‐h timepoint, pTau181 levels were higher when measured with Simoa (+13%, *p* = 0.002; 72 h: +14%, p = 0.002), and pTau217 levels were higher when measured with MSD (+14%, *p* < 0.001; 72 h: +16%, *p* < 0.001). pTau181 levels measured with Lumipulse and pTau217 levels measured with Lumipulse, Simoa, and LC‐MS were stable up until the 72‐h timepoint (i.e., the last timepoint assessed), as were LC‐MS npTau217 and %pTau217.

### 3.5 Effect of centrifugation settings

Varying the centrifugation duration (10 or 30 min) and centrifugation temperature (RT or 4°C) did not impact the measured K_2_EDTA plasma pTau181 and pTau217 levels (Figure 2.5). There was one exception of an inconsistent effect of centrifugation setting on pTau181 levels when measured with Lumipulse: Compared to 10 min RT centrifugation, pTau181 was higher with 10‐min 4°C centrifugation (+10%, *p* = 0.002), similar with 30‐min 4°C centrifugation, and lower with 30‐min RT centrifugation (−11%, *p* < 0.001).

### 3.6 Effect of transferring plasma to novel tubes

Compared to no transfers, up to four transfers of K_2_EDTA plasma to fresh polypropylene tubes using fresh polypropylene pipette tips did not alter the measured pTau181, pTau217, and npTau217 levels with any of the assays (Figure 2.6).

### 3.7 Effect of delayed −80°C freezing of plasma aliquots

The effect of delayed −80°C freezing of plasma aliquots after centrifugation while they were kept at RT, in the fridge (2°C to 8°C), or at −20°C is illustrated in Figures 2.7, 2.8, 2.9 and 2.10.

When plasma aliquots were kept at RT after centrifugation pending −80°C freezing, pTau181 and pTau217 levels remained stable up until the 2‐week timepoint (i.e., the last timepoint assessed), independent of the assay used for the measurements. LC‐MS npTau217 levels were lower at the 2‐week timepoint compared to direct −80°C freezing (−17%, *p* < 0.001), while LC–MS %pTau217 was not significantly changed at the 2‐week timepoint.

When plasma aliquots were kept in the fridge pending −80°C freezing, pTau181 levels remained stable up until the 2‐week timepoint independent of the assay used. For pTau217, levels were higher at the 1‐ and 2‐week timepoints when measured with MSD (1 week: +18%; 2 weeks: +19%; both *p* = 0.001), while they remained stable up until the 2‐week timepoint when measured with Lumipulse, Simoa, or LC‐MS. LC‐MS npTau217 and %pTau217 levels also remained stable up until the 2‐week timepoint.

Temporary −20° freezing pending −80°C storage up to 6 months (2 weeks, 2 months, 6 months; Figure 2.10) did not alter the measured levels of pTau181, pTau217, and npTau217, nor %pTau217, independent of the assay used.

### 3.8 Effect of applying freeze‐thaw (FT) cycles

Applying up to four additional FT cycles generally did not affect the pTau181, pTau217, and npTau217 levels, nor pTtau217%, with any of the assays (Figure 2.11), except there was an inconsistent decrease in pTau217 at +2FT when measured with Lumipulse (−11%, *p* = 0.01) and an inconsistent decrease in %pTau217 on LC‐MS at +1FT (+17%, *p* < 0.05), where both were unaffected in the more extreme conditions (up to +4FT).

### 3.9 Effect of pre‐analytical variations on other blood‐based biomarkers

**FIGURE 3:**
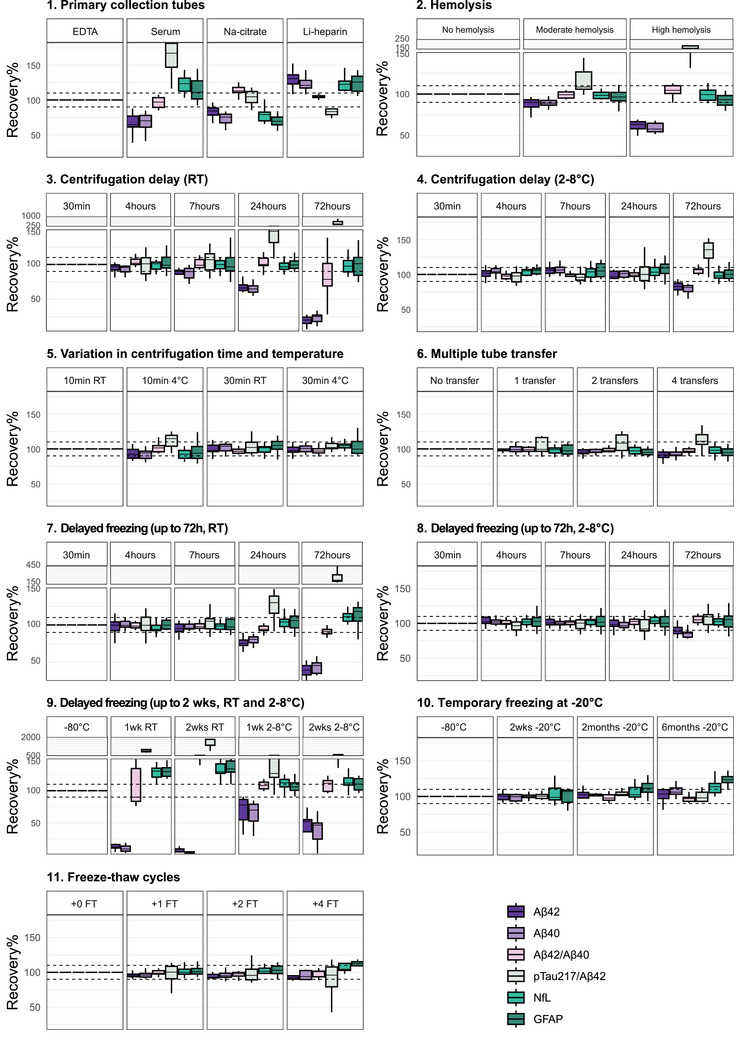
Recovery% plots visualizing the effect of 11 pre‐analytical variations on the levels of Aβ42, Aβ40, NfL, GFAP, and Aβ42/40 and pTau217/Aβ42 ratios as measured with Simoa HDX (pTau217 in the ratio, measured with Lumipulse). Levels measured in experimental conditions were normalized to levels measured in the reference conditions, i.e., centrifugation of K_2_EDTA blood tubes at 1800 × *g* at RT after standing at RT for 30 min, followed by plasma separation and aliquoting in low binding polypropylene screw‐capped storage tubes and storage at −80°C without any delays. Horizontal dashed lines at 90% and 110% represent an allowable 10% change in the experimental conditions compared to the reference condition. Box plots represent the median and interquartile range (IQR), whiskers represent the extreme data points that are within 1.5 times the IQR. Aβ42, beta amyloid protein (1‐42); Aβ40, beta amyloid protein (1‐40); EDTA, ethylene diamine tetraacetic acid; FT, freeze‐thaw; GFAP, glial fibrillary acidic protein; Li‐heparin, lithium‐heparin; Na‐citrate, sodium‐citrate; NfL, neurofilament light chain; RT, room temperature; Simoa, Single molecule array.

The results of the 11 pre‐analytical experiments on Aβ42, Aβ40, GFAP, and NfL levels and on the Aβ42/Aβ40 and pTau217/Aβ42 ratios are presented in Figure 3 and Supplementary File 1 and are described in detail below.

### 3.10 Effect of different primary collection tubes

Similar to results obtained for pTau, measured levels of Aβ42, Aβ40, GFAP, and NfL differed depending on the primary collection tube used for blood withdrawal (Figure 3.1).

In serum compared to K_2_EDTA plasma, both Aβ42 and Aβ40 were measured at lower levels (−36% and −30%, respectively; both *p* < 0.007). This primary collection tube effect could be mitigated by taking the Aβ42/Aβ40 ratio (Recovery%: 97%), but not by taking the pTau217/Aβ42 ratio (+67%, *p* = 0.001). GFAP and NfL were significantly higher in serum compared to K_2_EDTA plasma (+11% and +23%, respectively; both *p* < 0.007). In Na‐citrate compared to K_2_EDTA plasma, all markers were measured at lower levels (range decrease: −16% (Aβ42) and −31% (GFAP); all *p* < 0.009). Here, using the Aβ42/Aβ40 ratio did not mitigate the primary collection tube effect (+13%, *p* < 0.001), while the pTau217/Aβ42 ratio did (Recovery%: 105%). In plasma Li‐heparin compared to K_2_EDTA plasma, all markers were measured at higher levels (range increase: +21% (Aβ40) and +31% (Aβ42); all *p* < 0.007). Applying the Aβ42/Aβ40 ratio mitigated the primary collection tube effect (Recovery%: 105%), while applying the pTau217/Aβ42 ratio did not (−17%, *p* = 0.02).

### 3.11 Effect of hemolysis

Compared to no hemolysis, moderate hemolysis at 100 mg/dL Hb and high hemolysis at 500 mg/dL Hb (Figure 3.2) resulted in lower levels of Aβ42 (moderate: −10%, high: −36%; both *p* < 0.001) and Aβ40 (moderate: −11%, high: −42%; both *p* < 0.001). The effect of moderate or high hemolysis was mitigated by taking the Aβ42/Aβ40 ratio (Recovery% moderate: 99%, Recovery% high: 104%), but a significant effect of high hemolysis remained visible on the pTau217/Aβ42 ratio (+65%, *p* < 0.001). Hemolysis did not alter the measured GFAP and NfL levels.

### 3.12 Effect of centrifugation delays

When K_2_EDTA tubes were kept at RT pending centrifugation (Figure 3.3), Aβ42 and Aβ40 levels were only stable up until the 4‐h centrifugation delay timepoint. Thereafter, a consistent effect was noted on the levels, with more pronounced decreases observed with increasing durations of storage delays (Aβ42: up to −80% at the 72‐h timepoint; Aβ40:, up to −75% at the 72‐h timepoint; all tested conditions *p* < 0.02). The effect of delayed centrifugation when the K_2_EDTA tubes were kept at RT could be mitigated by taking the Aβ42/Aβ40 ratio (Recovery% at 72 h: −21%, *p* = 0.100). The pTau217/Aβ42 ratio was affected from the 24‐h timepoint onward (24 h: +48%; 72 h: +326%; both: *p* < 0.001).

At 2°C to 8°C, the levels of most BBMs appeared more stable than at RT. Precisely, the single Aβ42 and Aβ40 levels, as well as the Aβ42/Aβ40, and pTau217/Aβ42 ratios remained stable up until the 24‐h timepoint (Figure 3.4) when the K_2_EDTA tubes were kept at 2°C to 8°C (fridge) pending centrifugation. At the 72‐h timepoint, Aβ42 and Aβ40 levels were decreased (−17% and −19%, respectively, both: *p* < 0.001). The Aβ42/Aβ40 ratio was not relevantly affected at the 72‐h timepoint when the K_2_EDTA tubes were kept in the fridge pending centrifugation (Recovery% 107%), but the pTau217/Aβ42 ratio was (+36%, *p* < 0.001).

GFAP and NfL levels remained stable upon delayed centrifugation, both when the K_2_EDTA tubes were kept at RT and when they were kept in the fridge (2°C to 8°C) pending centrifugation.

### 3.13 Effect of centrifugation settings

Varying the centrifugation duration (10 or 30 min) and temperature (RT or 4°C) did not influence the measured levels of Aβ42, Aβ40, Aβ42/Aβ40 ratio, GFAP, or NfL (Figure 3.5). Only an inconstant effect of the 10 min 4°C centrifugation setting compared to 10 min RT centrifugation was observed on the pTau217/Aβ42 ratio (+15%, *p* < 0.001).

### 3.14 Effect of transferring plasma to novel tubes

Compared to no transfers, up to four transfers of plasma to fresh polypropylene tubes using fresh polypropylene pipette tips did not alter the measured levels of Aβ42, Aβ40, GFAP, or NfL, or the Aβ42/Aβ40 ratio (Figure 3.6). The pTau217/Aβ42 ratio was higher in the four‐time transfer condition compared to the no‐transfer condition (+11%, *p* = 0.003).

### 3.15 Effect of delayed −80°C freezing of plasma aliquots after centrifugation

The effect of delayed −80°C freezing, during which plasma aliquots are kept at different temperatures (RT, 2°C to 8°C, and −20°C), is illustrated in Figures 3.7, 3.8, 3.9, and 3.10.

Aβ42 and Aβ40 levels remained stable up until the 7‐h timepoint when plasma aliquots were kept at RT pending −80°C freezing, whereafter levels consistently decreased with increasing −80°C freezing delays until levels were decreased by 95% (Aβ42) and 97% (Aβ40) at the 2‐week timepoint. Although the Aβ42/Aβ40 ratio was still unchanged at the 2‐week RT‐condition timepoint (+82%, but *p* = 0.500), the decreases in the single analyte levels approached 0 pg/mL. At 2°C to 8°C, Aβ42 and Aβ40 levels in the plasma aliquots remained stable up until the 24‐h timepoint. After 2 weeks, however, levels were decreased by 48% (Aβ42; *p* < 0.001) and 54% (Aβ40; *p* < 0.001). The Aβ42/Aβ40 ratio was still unchanged at this 2‐week timepoint (R% +12%, *p* = 0.451). The pTau217/Aβ42 ratio was only stable up until the 7‐h timepoint when aliquots were kept at RT pending −80°C freezing, or up to the 72‐h timepoint when aliquots were kept in the fridge pending −80°C freezing.

GFAP and NfL levels remained stable up until the 24‐h timepoint when the plasma aliquots were kept at RT pending −80°C freezing. At the 72‐h timepoint, levels of both markers were increased (GFAP: +18%, NfL: +11%; both: *p* < 0.003) and continued to increase up until the 1‐week timepoint (GFAP: 1 week +30%, 2 weeks +34%; NfL: 1 week +31%, 2 weeks +30%; all: *p* < 0.001). For both GFAP and NfL, levels were measured at similar levels in plasma aliquots that were kept in the fridge pending −80°C freezing. GFAP was increased relevantly at the 2‐week timepoint (+10%, *p* = 0.003) and NfL at the 1‐week timepoint (+12%, *p* = 0.002; 2 weeks: +12%, *p* < 0.001).

Freezing plasma aliquots at −20°C pending −80°C storage did not affect the measured levels of Aβ42 and Aβ40, or the Aβ42/Aβ40 ratio, and pTau217/ Aβ42 ratios, up until the latest timepoint assessed, 6 months. However, the levels of both GFAP and NfL increased upon temporary −20°C storage. The levels were unchanged at the 2‐week timepoint, but at the 2‐month timepoint, GFAP levels were increased (+12%, *p* = 0.002; 6 months: +24%, *p* < 0.001), and at the 6‐month timepoint NfL levels were also increased (+14%, *p* = 0.003).

### 3.16 Effect of applying freeze‐thaw cycles

Applying up to four additional FT cycles did not alter the measured levels of Aβ42, Aβ40, and NfL, or the Aβ42/Aβ40 and pTau217/Aβ42 ratios. GFAP was slightly increased at the fourth FT cycle (+13%; no statistical testing was performed for condition 4 (4 FT) because of its limited *n* = 5; Figure 3.11).

### 3.17 Established workflow

**FIGURE 4:**
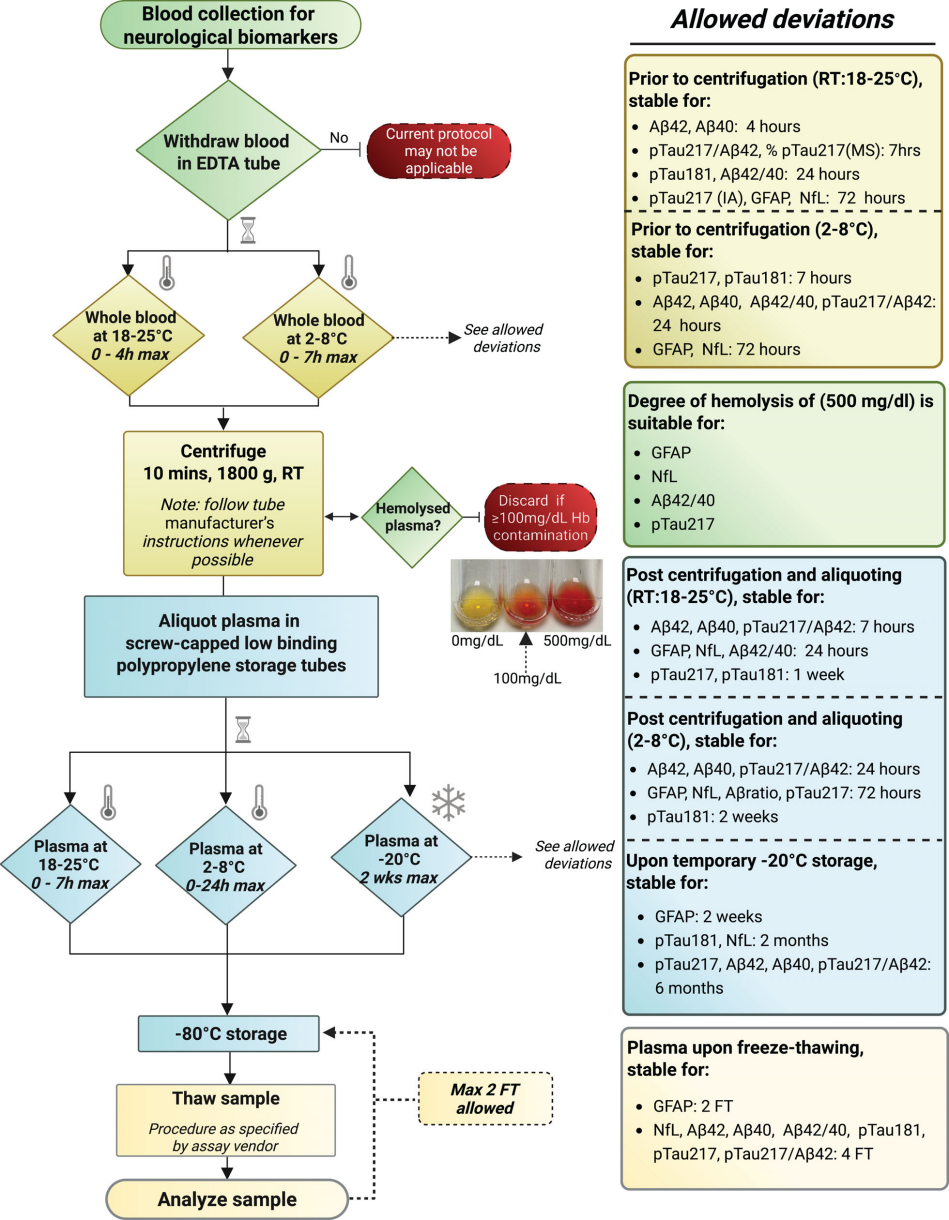
Evidence‐based sample handling protocol for accurate neurological blood‐based biomarker (BBMs) measurement. The flowchart is tailored to mitigate any pre‐analytical effect on the most sensitive neurological biomarker. When measuring a specific marker, more flexibility at certain steps might be possible, which can be derived from the “allowed deviations” column. Of note, the advised, optimal sample handling protocol includes a total processing time of less than 2 h. Hemolysis degree can be measured or visually inspected using the color grade presented in the figure or by comparing it to the commonly used hemolysis palettes.^45^ EDTA, ethylene diamine tetraacetic acid; FT, freeze‐thaw; Hb, hemoglobin; max, maximum; RT, room temperature.

The presented findings, together with the workgroup's suggestions, are summarized as an evidence‐based, consensus sample handling protocol in Figure 4. This protocol visualizes our recommendations for EDTA plasma sample handling to ensure reliable quantification of neurological BBMs upon clinical adoption. The sample handling protocol is presented as a flowchart, in which each step in the pre‐analytical phase is depicted. To mitigate the effects of pre‐analytical variability, each step was tailored to the biomarker that was most susceptible to variation at that step. For the more stable BBMs, the protocol includes *allowed deviations*, reflecting their demonstrated stability under specific handling conditions.

## 4 DISCUSSION

In this SABB–GBSC workgroup study, endorsed by the Alzheimer's Association, we extensively evaluated the impact of pre‐analytical sample handling variations on key neurological plasma markers measured with different technologies and assays, aiming to establish an evidence‐based sample handling protocol that mitigates pre‐analytical effects. We systematically varied conditions spanning the entire pre‐analytical phase, mimicking real‐world laboratory practices. According to our results, critical steps in the pre‐analytical sample handling workflow requiring most strict standardization are the type of primary collection tube used to withdraw blood and delays until centrifugation and −80°C freezing. We observed that Aβ42, Aβ40, and pTau217/Aβ42 were most sensitive to pre‐analytical sample handling variations. The Aβ42/40 ratio, pTau217, %pTau217 (LC‐MS), pTau181, GFAP, and NfL showed better stability. We summarized our findings in a sample handling workflow, designed for real‐world feasibility by including controlled flexibility in sample processing without compromising biomarker accuracy. While optimal guidelines^24^, ^25^ would limit total collection‐to‐freezer time to 2 h, its feasibility is limited to controlled settings. Decentralized (e.g., general practitioners) and resource‐limited settings (e.g., low/middle‐income countries) require flexibility. Following the key priority of the World Health Organization for global access to AD‐related BBMs for equitable healthcare,^26^ the global feasibility of guidelines is essential.

Varying the primary collection tube resulted in pronounced differences in BBM levels for all assessed markers, in agreement with previous literature.^11^, ^12^, ^14^, ^19^, ^27^, ^28^, ^29^ According to our comparative pTau results, the effect of the primary collection tube on the measured BBM level is assay‐dependent. We also observed this when comparing pre‐analytics on Aβ42 and Aβ40 levels measured with various technologies.^11^ Assay dependency is likely explained by differences in detection methodologies and assay components. Despite differences in measured BBM levels across assays, technologies,^30^, ^31^ and sample matrices (serum and different kinds of plasma),^11^, ^12^, ^14^, ^19^, ^27^, ^28^, ^29^, ^32^ previous studies reported a similar diagnostic performance of plasma and serum pTau measurements in reflecting amyloid pathology.^27^, ^32^, ^33^ Nevertheless, upon clinical implementation, we recommend avoiding interchangeable use of different blood sample matrices, since each matrix asks for its own clinical cutoffs and normal reference ranges.

We recommend centrifuging K_2_EDTA plasma tubes promptly without any delays, but based on our data, a maximum of 4‐h delay is allowed if K_2_EDTA plasma collection tubes are kept at RT or 7 h if they are kept refrigerated. These recommendations were tailored to the most unstable markers before centrifugation, i.e., Aβ42 and Aβ40 at RT and pTau181 and pTau217 at 2°C to 8°C. Our findings are consistent with previous studies.^11^, ^12^, ^13^, ^19^, ^34^ Pre‐centrifugation instability of Aβ might be explained by proteolytic degradation, since supplementing samples with protease inhibitors was shown to extend the stability of Aβ42 and Aβ40.^35^ Interestingly, adding protease inhibitors did not extend the stability of pTau, NfL, and GFAP,^35^ suggesting that specifically Aβ measurements are susceptible to protease activity. Proteolytic enzymes such as trypsin primarily target lysine and arginine residues.^36^ Since Aβ proteins contain two lysine and one arginine residue, this might explain their susceptibility to proteolytic degradation. Interestingly, rates of decline over centrifugation delay were relatively similar for the Aβ42 and Aβ40 peptides. As a result, the instability of Aβ can mostly be mitigated by applying the Aβ42/40 ratio. Note that applying the pTau217/Aβ42 ratio does not mitigate pre‐analytical effects, because pre‐analytical effects are different for pTau and Aβ42. While well‐controlled studies showed that this ratio outperformed pTau217 alone as a diagnostic biomarker for AD pathophysiology,^37^, ^38^, ^39^ its reliability seems to be compromised in real‐world settings without stringent sample handling. It was an unexpected finding that pTau181 and pTau217 levels pre‐centrifugation were stable longer at RT than refrigerated. Pre‐centrifugation instability of pTau seemed, however, assay‐dependent, as is generally the case with most assays; stable levels were measured across the 2°C to 8°C centrifugation delay conditions. The negatively charged phosphate groups as present in pTau proteoforms form salt bridges with the protonated side chains of lysine and arginine residues, obscuring the proteolytic action of proteases.^40^ This might make pTau less prone to proteolytic degradation. Supporting this, earlier pre‐analytical studies that measured both total tau (i.e., a mix of npTau and pTau) and pTau specifically confirmed the higher pre‐analytical stability of pTau compared to total tau measurements.^11^, ^13^ GFAP and NfL levels were resistant to pre‐centrifugation delays up to 72 h at RT and 2°C to 8°C, in agreement with previous literature.^11^, ^12^

After centrifugation and plasma separation, longer delays until sample freezing can be allowed. Again, our recommendation is not to introduce any delays at this step, but based on our results, we are able to allow freezing delays of up to 7 h when plasma is kept at RT and up to 24 h when plasma is kept cold. Aβ42 and Aβ40 were again the most susceptible markers driving our recommendation. The Aβ42 and Aβ40 levels consistently decreased with increasing freezing delays, until almost undetectable levels were reached after 2 weeks at RT. Of note, degradation of Aβ42 and Aβ40 is slower in separated plasma compared to whole blood, likely due to the lower concentration of proteolytic enzymes in plasma. The Aβ42/40 ratio, GFAP, and NfL were stable for up to 24 h at RT and 72 h at 2°C to 8°C. pTau217, %p‐Tau217, and pTau181 remained stable for up to 1 week at RT and up to 72 h at 2°C to 8°C, independent of the assay used. The findings are consistent with previous findings.^11^, ^13^, ^18^, ^34^

Other pre‐analytical variations assessed in the current study are sample hemolysis, centrifugation settings, plasma transferring, temporary −20°C freezing, and freeze‐thawing. Sample hemolysis can occur, for example, due to prolonged tourniquet duration^41^, ^42^ and occurs in up to 3.3% of routine blood collections,^43^ and more often in the elderly population.^44^ We found that a moderate degree of hemolysis of 100 mg/dL hemoglobin^45^ already influenced the levels of Aβ42, Aβ40, pTau217/Aβ42, and, to a lesser extent, pTau181. Similar trends were observed in a previous study.^46^ Therefore, moderately hemolyzed samples should be discarded *or* interpreted with caution. Immunoassays may suffer more from hemolysis than MS assays, as antibody‐based assays can cross‐react with free hemoglobin. We recommend discarding samples with a moderate to high hemolysis degree. There was no effect of centrifugation settings on the BBM measurements in our study, so we recommend following standard protocols and/or manufacturer's guidelines. Also, tube transferring had no impact on the assessed BBM levels, suggesting that the assessed proteins in plasma did not adsorb to lab plastics tested here, so no stringent recommendations for this step were made. We noted an impact of −20°C storage on GFAP and NfL levels, in line with previous work.^47^ GFAP levels were increased at the 2‐month timepoint, and NfL levels were increased at 6 months. For long‐term biobanking, we thus recommend storing samples at −80°C and avoiding prolonged (>2 weeks) −20°C storage. Freezing and thawing (up to four times) of plasma samples minimally impacted most BBMs, as reported earlier.^11^, ^12^ Only GFAP levels were slightly increased at the fourth FT cycle. Hence, our recommendation is to limit freeze‐thawing of K_2_EDTA plasma samples to two cycles. It is interesting that both GFAP and NfL levels tended to increase at −20°C storage, with GFAP also showing increases with freeze‐thawing, especially because these were the conditions in which Aβ and pTau were stable. This suggests that a different mechanism results in protein instability for GFAP and NfL than for Aβ and pTau. Both GFAP and NfL exhibit structural and conformational flexibility depending on the surrounding matrix and are known to form higher‐order assemblies.^48^, ^49^ For example, GFAP adopts an open conformation in artificial CSF compared to PBS due to decreased hydrogen bonding. This might also happen in samples exposed to lower temperature freezing or freeze‐thawing, exposing more epitopes to antibodies, and, hence, increase its measurability with immunoassays.^48^ This hypothesis can be tested with MS‐based experiments.

A key strength of this study is its extensive evaluation of pre‐analytical factors, encompassing many conditions as they occur in daily practice, and tested on the most promising AD BBMs using both immunoassays and LC‐MS assays. Although some assays exhibited relatively large variability across pre‐analytical conditions, including several assays to test the same biomarker increased the generalizability of our findings. The inclusion of both healthy and AD participants covered the clinically relevant range of biomarker measurements. In addition, we generated consensus among experts in the field, enhancing clinical adoption of the developed guideline. Among our study's limitations are (1) focusing on K_2_EDTA (for a serum‐based protocol, refer to van Lierop et al.^28^), (2) inability to simulate all pre‐analytical conditions (e.g., transport), (3) assuming BBM stability at ‐80°C without experimental verification, (4) not assessing fresh unfrozen samples, (5) not including demographic and clinical information while not knowing whether those could confound pre‐analytical effects on BBM measurements, and (6) not being able to include all possible pTau assays/technologies due to our limited sample volume per experiment/condition. The establishment of a new biorepository including more handling factors is needed, which also offers the possibility to assess pre‐analytics of novel markers, such as plasma brain derived tau (BD‐tau), eMTBR243‐tau, beta‐synuclein, and SNAP‐25,^50^, ^51^, ^52^, ^53^ and to include FDA‐approved and/or in vitro diagnositic medical devices regulations (IVDR)‐certified assays for the markers assessed here, once these become available, in addition to exploring whether demographic or clinical variables would modify the magnitude of pre‐analytical effects, and test for longer‐term storage at −20°C

To conclude, this study provides an evidence‐based, consensus sample handling protocol that ensures reliable neurological biomarker measurement upon clinical adoption. The protocol has flexibility and can be easily tailored to particular BBMs, addressing the important aspect of global BBM accessibility and healthcare equity. The presented standardized handling protocol will improve BBM reliability for diagnostic, prognostic, and disease monitoring approaches in clinical studies and upon routine clinical implementation.

## CONFLICT OF INTEREST STATEMENT

M.G., D.A.B., M.vL., B.B., I.H., and S.J. have nothing to disclose. IV has consultancy contracts with Quanterix and Neurogen Biomarking, which are paid directly to her institution. I.V. performs contract research for Nitrase Therapeutics and Roche Diagnostics, which is paid directly to her institution. W.F. has been funded by ZonMW, NWO, EU‐JPND, EU‐IHI, Alzheimer Nederland, Hersenstichting CardioVascular Onderzoek Nederland, Health Holland, Topsector Life Sciences & Health, stichting Dioraphte, Noaber Foundation, Pieter Houbolt Fonds, Gieskes‐Strijbis fonds, stichting Equilibrio, Edwin Bouw fonds, Pasman stichting, Philips, Biogen MA Inc., Novartis‐NL, Life‐MI, AVID, Roche BV, Lilly Nederland, Fujifilm, Eisai, Combinostics. W.F. holds the Pasman Chair. W.F. is the recipient of ABOARD, which is a public–private partnership receiving funding from ZonMW (No. 73305095007) and Health Holland, Topsector Life Sciences & Health (PPP‐allowance; No. LSHM20106) and is the recipient of TAP‐dementia (www.tap‐dementia.nl), receiving funding from ZonMw (No. 10510032120003). TAP‐dementia receives co‐financing from Avid Radiopharmaceuticals, Roche, and Amprion. W.F. is recipient of IHI‐PROMINENT (No. 101112145) and IHI‐AD‐RIDDLE (No. 101132933). PROMINENT and AD‐RIDDLE are supported by the Innovative Health Initiative Joint Undertaking (IHI JU). The JU receives support from the European Union's Horizon Europe research and innovation programme and COCIR, EFPIA, EuropaBio, MedTech Europe, and Vaccines Europe, with Davos Alzheimer's Collaborative, Combinostics OY, Cambridge Cognition Ltd., C2N Diagnostics LLC, and neotiv GmbH. All funding is paid to her institution. W.F. has been an invited speaker at Biogen MA Inc., Danone, Eisai, WebMD Neurology (Medscape), NovoNordisk, Springer Healthcare, and European Brain Council. All funding is paid to her institution. W.F. is consultant to Oxford Health Policy Forum CIC, Roche, Biogen MA Inc., Eisai, Eli Lilly, Owkin France, Nationale Nederlanden Ventures. All funding is paid to her institution. W.F. participated in advisory boards of Biogen MA Inc., Roche, and Eli Lilly, is a member of the steering committee of phase 3 EVOKE/EVOKE+ studies (NovoNordisk), and is a member of the steering committee of the phase 3 Trontinemab study (Roche). All funding is paid to her institution. In addition, W.F. is a member of the steering committee of PAVE and Think Brain Health, chair of the Scientific Leadership Group of InRAD, a former associate editor of Alzheimer, Research & Therapy in 2020/2021, associate editor at Brain, and a member of the supervisory board (Raad van Toezicht) of Trimbos Instituut. O.H. is an employee of Lund University and Eli Lilly. N.LB. and M.V. are employees of Fujirebio Europe N.V. J.H. and L.H. are employees and shareholders of ALZpath. K.K. and P.V. are salaried employees at C2N Diagnostics and receive compensation from the company in the form of salary and/or equity. K.B. has served as a consultant and on advisory boards for AbbVie, AC Immune, ALZPath, AriBio, Beckman‐Coulter, BioArctic, Biogen, Eisai, Lilly, Moleac Pte. Ltd., Neurimmune, Novartis, Ono Pharma, Prothena, Quanterix, Roche Diagnostics, Sanofi, and Siemens Healthineers; has served on data monitoring committees for Julius Clinical and Novartis; has given lectures, produced educational materials, and participated in educational programs for AC Immune, Biogen, Celdara Medical, Eisai, and Roche Diagnostics; and is a co‐founder of Brain Biomarker Solutions in Gothenburg AB (BBS), which is a part of the GU Ventures Incubator Program, outside the work presented in this paper. H.Z. has served on scientific advisory boards and/or as a consultant for AbbVie, Acumen, Alector, Alzinova, ALZpath, Amylyx, Annexon, Apellis, Artery Therapeutics, AZTherapies, Cognito Therapeutics, CogRx, Denali, Eisai, Enigma, LabCorp, Merck Sharp & Dohme, Merry Life, Nervgen, Novo Nordisk, Optoceutics, Passage Bio, Pinteon Therapeutics, Prothena, Quanterix, Red Abbey Labs, reMYND, Roche, Samumed, ScandiBio Therapeutics AB, Siemens Healthineers, Triplet Therapeutics, and Wave; has given lectures sponsored by Alzecure, BioArctic, Biogen, Cellectricon, Fujirebio, LabCorp, Lilly, Novo Nordisk, Oy Medix Biochemica A.B., Roche, and WebMD; and is a co‐founder of Brain Biomarker Solutions in Gothenburg AB (BBS), which is a part of the GU Ventures Incubator Program (outside submitted work). R.M.E. and E.A.M. are full‐time employees of the Alzheimer's Association. C.E.T. has research contracts with Acumen, ADx Neurosciences, AC‐Immune, Alamar, Aribio, Axon Neurosciences, Beckman‐Coulter, BioConnect, Bioorchestra, Brainstorm Therapeutics, C2N diagnostics, Celgene, Cognition Therapeutics, EIP Pharma, Eisai, Eli Lilly, Fujirebio, Instant Nano Biosensors, Merck, Muna, Novo Nordisk, Olink, PeopleBio, Quanterix, Roche, Toyama, Vaccinex, and Vivoryon. C.E.T. is editor in chief of *Alzheimer Research and Therapy*, serves on the editorial boards of *Molecular Neurodegeneration*, *Alzheimer's & Dementia*, *Neurology: Neuroimmunology & Neuroinflammation*, and *Medidact Neurologie*/Springer, and is a member of the committee to define guidelines for cognitive disturbances and a committee for acute neurology in the Netherlands. C.E.T. has consultancy/speaker contracts for Aribio, Biogen, Beckman‐Coulter, Cognition Therapeutics, Danaher, Eisai, Eli Lilly, Janssen, Merck, Novo Nordisk, Novartis, Olink, Roche, Sanofi, and Veravas. Author disclosures are available in the Supporting Information.

## CONSENT STATEMENT

All human subjects provided informed consent.

## Supporting information



Supporting Information

Supporting Information
