# Supplementary material for: Evidence‐based standardized sample handling protocol for accurate blood‐based Alzheimer's disease biomarker measurement: Results and consensus of the Global Biomarker Standardization Consortium
Source: Alzheimers Dement. 2025 Sep 30;21(10):e70752. doi: 10.1002/alz.70752 (PMC12481164; doi:10.1002/alz.70752)
Supplement: Supplementary file 2 — Supporting Information [file ALZ-21-e70752-s002.pdf]

Experimental Variation (Condition)	Median R% (IQR)	%Change from median R%	P_value	Median R% (IQR)	%Change from median R%	P_value	Median R% (IQR)	%Change from median R%	P_value	Median R% (IQR)	%Change from median R%	P_value	Median R% (IQR)	%Change from median R%	P_value	Median R% (IQR)	%Change from median R%	P_value
	**Aβ40**			**Aβ42**			**Aβ42/Aβ40**			**GFAP**			**NFL**			**pTau217/Aβ42**		
Reference	100 (100-100)	0		100 (100-100)	0		100 (100-100)	0		100 (100-100)	0		100 (100-100)	0		100 (100-100)	0	
Cent Delay at RT (4 hrs)	96 (89-98)	-4	0.000	97 (92-99)	-3	0.173	102 (100-108)	2	0.078	99 (95-110)	-1	0.903	101 (93-103)	1	0.670	101 (87-110)	1	0.893
Cent Delay at RT (7 hrs)	90 (81-94)	**-10.3**	0.000	90 (86-92)	**-10.5**	**0.013**	99 (95-107)	-1	0.855	97 (91-110)	-3	0.855	100 (94-106)	0	1.000	107 (92-115)	7	0.216
Cent Delay at RT (24 hrs)	65 (60-69)	**-35**	0.000	66 (63-71)	**-34**	**0.000**	100 (98-109)	0	0.542	99 (94-105)	-1	0.855	97 (93-102)	-3	0.268	148 (131-163)	**48**	**0.000**
Cent Delay at RT (72 hrs)	25 (18-27)	**-75**	0.000	20 (15-24)	**-80**	**0.001**	79 (69-102)	**-21**	0.102	101 (84-110)	1	0.903	98 (89-106)	-2	0.502	426 (379-684)	**326**	**0.001**
Reference	100 (100-100)	0		100 (100-100)	0		100 (100-100)	0		100 (100-100)	0		100 (100-100)	0		100 (100-100)	0	
Cent Delay in the fridge (4 hrs)	104 (102-109)	4	0.004	102 (97-105)	2	0.229	98 (94-100)	-2	**0.048**	106 (101-108)	6	**0.001**	105 (99-107)	5	**0.008**	98 (89-103)	-2	0.252
Cent Delay in the fridge (7 hrs)	106 (103-110)	6	0.001	106 (102-109)	6	**0.002**	100 (97-101)	0	0.252	105 (97-115)	5	**0.041**	104 (96-108)	4	0.277	96 (92-100)	-4	0.330
Cent Delay in the fridge (24 hrs)	101 (96-105)	1	0.804	97 (94-105)	-3	0.639	99 (97-102)	-1	0.454	109 (101-115)	9	**0.022**	103 (99-110)	3	0.083	100 (91-111)	0	0.847
Cent Delay in the fridge (72 hrs)	81 (75-84)	**-19**	0.000	83 (78-88)	**-17**	**0.000**	107 (102-108)	7	**0.000**	100 (94-106)	0	0.847	98 (95-103)	-2	0.389	136 (123-145)	**36**	**0.000**
Reference	100 (100-100)	0		100 (100-100)	0		100 (100-100)	0		100 (100-100)	0		100 (100-100)	0		100 (100-100)	0	
Storage at RT (4hr)	99 (97-105)	-1	0.934	100 (92-104)	0	0.847	98 (96-103)	-2	0.389	100 (95-106)	0	0.847	94 (92-100)	-6	0.083	100 (93-110)	0	1.000
Storage at RT (7 hrs)	96 (95-101)	-4	0.073	95 (91-101)	-5	**0.022**	98 (95-102)	-2	0.107	97 (94-108)	-3	0.890	99 (94-101)	-1	0.229	105 (95-108)	5	0.670
Storage at RT (24 hrs)	81 (76-84)	**-19**	**0.000**	75 (72-80)	**-25**	**0.000**	94 (92-98)	-6	**0.002**	106 (96-112)	6	0.229	104 (98-108)	4	0.135	130 (116-139)	**30**	**0.000**
Storage at RT (72 hrs)	46 (35-49)	**-54**	**0.000**	39 (33-45)	**-61**	**0.000**	91 (88-94)	-9	**0.000**	118 (105-123)	**18**	**0.003**	111 (105-115)	**11**	**0.000**	219 (203-306)	**119**	**0.000**
Reference	100 (100-100)	0		100 (100-100)	0		100 (100-100)	0		100 (100-100)	0		100 (100-100)	0		100 (100-100)	0	
storage at 4C 4 hrs	102 (99-105)	2	0.169	102 (100-109)	2	0.083	100 (99-104)	0	0.524	103 (96-108)	3	0.252	102 (98-106)	2	0.252	97 (91-102)	-3	0.173
storage at 4C 7 hrs	100 (98-103)	0	0.639	101 (98-106)	1	0.359	101 (98-103)	1	0.359	102 (96-110)	2	0.561	104 (98-105)	4	0.107	100 (93-105)	0	0.715
storage at 4C 24 hrs	97 (94-101)	-3	0.208	98 (96-105)	-2	0.804	103 (98-106)	3	0.188	100 (95-110)	0	0.421	103 (100-109)	3	**0.048**	92 (89-105)	-8	0.542
storage at 4C 72 hrs	86 (80-87)	**-14**	**0.000**	89 (85-94)	**-11**	**0.003**	105 (101-110)	5	**0.035**	106 (95-111)	6	0.252	103 (98-107)	3	0.277	109 (98-113)	9	0.057
Reference	100 (100-100)	0		100 (100-100)	0		100 (100-100)	0		100 (100-100)	0		100 (100-100)	0		100 (100-100)	0	
Temporary storage for 1 wk at RT	9 (6-14)	**-91**	**0.000**	12 (11-16)	**-88**	**0.031**	90 (83-135)	**-10.1**	1.000	130 (122-136)	**30**	**0.000**	131 (121-135)	**31**	**0.000**	703 (609-856)	**603**	**0.031**
Temporary storage for 2 wk at RT	3 (3-5)	**-97**	**0.008**	5 (5-9)	**-95**	0.250	182 (161-203)	**82**	0.500	134 (129-147)	**34**	**0.000**	130 (127-142)	**30**	**0.000**	2000 (1348-2019)	**1900**	0.250
Temporary storage for 1 wk at 2-8°C	70 (53-79)	**-30**	**0.000**	78 (55-86)	**-22**	**0.000**	108 (103-113)	8	0.055	106 (101-112)	6	**0.018**	112 (106-118)	**12**	**0.002**	127 (121-191)	**27**	**0.000**
Temporary storage for 2 wk at 2-8°C	46 (24-50)	**-54**	**0.000**	52 (36-55)	**-48**	**0.000**	112 (99-116)	**12**	0.463	110 (101-119)	**10.3**	**0.003**	112 (109-120)	**12**	**0.000**	210 (176-290)	**110**	**0.000**
Reference	100 (100-100)	0		100 (100-100)	0		100 (100-100)	0		100 (100-100)	0		100 (100-100)	0		100 (100-100)	0	
temporary storaget at -20°C (2wk)	100 (94-103)	0	0.455	100 (95-103)	0	0.839	100 (98-102)	0	0.946	107 (92-109)	7	0.542	99 (95-112)	-1	0.497	100 (97-103)	0	0.970
temporary storaget at -20°C (2 mons)	102 (101-103)	2	0.094	102 (98-106)	2	0.376	98 (95-102)	-2	0.273	112 (106-118)	**12**	**0.002**	102 (99-113)	2	0.057	103 (102-106)	3	0.176
temporary storaget at -20°C(6 mons)	106 (103-112)	6	**0.005**	103 (97-110)	3	0.273	97 (93-99)	-3	**0.008**	124 (119-128)	**24**	**0.000**	114 (105-118)	**14**	**0.003**	98 (93-106)	-2	0.910
Reference	100 (100-100)	0		100 (100-100)	0		100 (100-100)	0		100 (100-100)	0		100 (100-100)	0		100 (100-100)	0	
Tube transfer 1x	99 (96-103)	-1	0.903	99 (97-100)	-1	0.104	99 (96-102)	-1	0.296	97 (92-106)	-3	0.463	99 (94-101)	-1	0.426	99 (96-116)	-1	0.414
Tube transfer 2x	96 (95-99)	-4	**0.020**	95 (93-99)	-5	**0.002**	98 (96-101)	-2	0.135	95 (92-99)	-5	**0.007**	97 (93-102)	-3	0.153	108 (98-120)	8	0.127
Tube transfer 4x	92 (90-95)	-8	**0.001**	91 (88-96)	-9	**0.000**	97 (95-100)	-3	0.104	95 (91-100)	-5	**0.025**	98 (94-103)	-2	0.391	111 (106-120)	**11**	**0.003**
Reference	100 (100-100)	0		100 (100-100)	0		100 (100-100)	0		100 (100-100)	0		100 (100-100)	0		100 (100-100)	0	
Centrifugation settings (10 min at 4°C)	94 (86-97)	-6	**0.001**	92 (87-99)	-8	**0.010**	101 (96-105)	1	0.639	94 (86-103)	-6	0.135	92 (87-98)	-8	**0.003**	115 (104-120)	**15**	**0.001**
Centrifugation settings (30 min at RT)	104 (97-107)	4	0.252	98 (96-105)	-2	0.762	97 (94-100)	-3	**0.030**	105 (100-111)	5	0.064	102 (95-103)	2	0.762	102 (94-110)	2	0.454
Centrifugation settings (30 min at 4°C)	101 (96-104)	1	0.978	98 (95-102)	-2	0.303	97 (94-100)	-3	0.073	100 (94-111)	0	0.359	105 (102-107)	5	**0.012**	102 (100-108)	2	0.169
Reference	100 (100-100)	0		100 (100-100)	0		100 (100-100)	0		100 (100-100)	0		100 (100-100)	0		100 (100-100)	0	
collection tube (Serum)	70 (61-78)	**-30**	**0.007**	64 (61-77)	**-36**	**0.002**	97 (89-103)	-3	0.169	111 (101-127)	**11**	**0.007**	123 (113-131)	**23**	**0.001**	167 (147-181)	**67**	**0.001**
collection tube (Na-Citrate)	75 (67-80)	**-25**	**0.008**	84 (78-88)	**-16**	**0.008**	113 (111-118)	**13**	**0.000**	69 (64-75)	**-31**	**0.001**	79 (70-83)	**-21**	**0.000**	105 (96-112)	5	0.414
collection tube (Li-Heparin)	121 (117-128)	**21**	**0.000**	131 (123-136)	**31**	**0.000**	105 (104-106)	5	**0.004**	126 (113-134)	**26**	**0.004**	122 (114-127)	**22**	**0.007**	83 (79-87)	**-17**	**0.021**
Reference	100 (100-100)	0		100 (100-100)	0		100 (100-100)	0		100 (100-100)	0		100 (100-100)	0		100 (100-100)	0	
FT (1X)	96 (93-98)	-4	**0.022**	95 (94-97)	-5	**0.003**	99 (97-102)	-1	0.847	101 (98-106)	1	0.389	100 (97-105)	0	0.847	100 (91-109)	0	0.808
FT (2X)	95 (93-99)	-5	**0.012**	93 (92-97)	-7	**0.000**	98 (95-100)	-2	**0.041**	103 (98-109)	3	0.277	102 (98-106)	2	0.389	96 (89-104)	-4	0.268
FT (4X)	94 (90-103)	-6	0.313	92 (90-95)	-8	0.125	101 (94-102)	1	0.813	113 (109-115)	**13**	0.313	104 (103-113)	4	0.125	96 (79-108)	-4	0.625
Reference	100 (100-100)	0		100 (100-100)	0		100 (100-100)	0		100 (100-100)	0		100 (100-100)	0		100 (100-100)	0	
hemolysis (Low)	89 (87-92)	**-11**	**0.012**	90 (84-93)	**-10**	**0.000**	99 (95-103)	-1	**0.041**	96 (92-102)	-4	0.277	98 (95-102)	-2	0.389	109 (106-127)	9	0.268
hemolysis (high)	58 (55-65)	**-42**	**0.000**	64 (58-67)	**-36**	**0.000**	104 (101-110)	4	0.068	93 (87-98)	-7	**0.002**	99 (93-105)	-1	0.808	165 (152-198)	**65**	**0.000**

Experimental Variation (condition)	Median R% (IQR)	%Change from median R%	P_value	Median R% (IQR)	%Change from median R%	P_value	Median R% (IQR)	%Change from median R%	P_value	Median R% (IQR)	%Change from median R%	P_value	Median R% (IQR)	%Change from median R%	P_value	Median R% (IQR)	%Change from median R%	P_value	Median R% (IQR)	%Change from median R%	P_value	Median R% (IQR)	%Change from median R%	P_value
	**pTau181 _ Lumipulse**			**pTau181_Simoa**			**pTau217_Lumipulse**			**pTau217_Simoa**			**pTau217_MSD**			**pTau217_MassSpec.**			**npTau217_MassSpec**			**pTau217/npTau217**		
Reference	100 (100-100)	0		100 (100-100)	0		100 (100-100)	0		100 (100-100)	0		100 (100-100)	0		100 (100-100)	0		100 (100-100)	0		100 (100-100)	0	
Cent Delay at RT (4 hrs)	88 (82-104)	**-12**	0.121	99 (94-105)	-1	0.934	95 (92-101)	-5	**0.026**	101 (98-102)	1	0.635	103 (95-106)	3	0.561	92 (75-103)	-8	0.250	100 (96-106)	0	0.847	87 (76-113)	**-13**	0.426
Cent Delay at RT (7 hrs)	93 (91-102)	-7	0.258	100 (95-107)	0	0.720	102 (91-104)	2	0.847	101 (93-103)	1	0.583	104 (97-114)	4	0.107	70 (63-104)	**-30**	0.203	101 (98-108)	1	0.489	68 (61-103)	**-32**	0.129
Cent Delay at RT (24 hrs)	97 (95-119)	-3	0.346	95 (89-99)	-5	**0.008**	99 (92-106)	-1	0.804	99 (91-101)	-1	0.153	102 (92-112)	2	0.489	75 (64-80)	**-25**	**0.027**	100 (97-104)	0	0.890	73 (65-82)	**-27**	**0.027**
Cent Delay at RT (72 hrs)	122 (103-149)	**22**	**0.012**	105 (100-123)	5	0.064	101 (93-108)	1	0.890	98 (93-106)	-2	0.670	96 (89-104)	-4	0.359	64 (58-78)	**-36**	**0.016**	112 (106-116)	**12**	**0.002**	71 (48-73)	**-29**	**0.016**
Reference	100 (100-100)	0		100 (100-100)	0		100 (100-100)	0		100 (100-100)	0		100 (100-100)	0		100 (100-100)	0		100 (100-100)	0		100 (100-100)	0	
Cent Delay in the fridge (4 hrs)	93 (88-95)	-7	**0.010**	100 (97-113)	0	0.182	99 (92-103)	-1	0.286	98 (95-105)	-2	0.934	106 (101-116)	6	**0.038**	109 (101-137)	9	0.109	99 (95-104)	-1	0.903	116 (100-130)	**16**	0.219
Cent Delay in the fridge (7 hrs)	92 (86-95)	-8	**0.000**	102 (98-105)	2	0.268	100 (94-108)	0	0.762	102 (99-108)	2	0.229	109 (103-114)	9	**0.011**	113 (97-136)	**13**	0.148	98 (93-109)	-2	1.000	111 (103-126)	**11**	0.109
Cent Delay in the fridge (24 hrs)	90 (86-93)	-9.9	**0.022**	113 (104-118)	**13**	**0.002**	98 (91-104)	-2	0.524	106 (98-110)	6	0.151	114 (104-126)	**14**	**0.001**	102 (88-112)	2	0.938	100 (92-110)	0	0.761	93 (85-111)	-7	0.938
Cent Delay in the fridge (72 hrs)	102 (97-117)	2	0.268	114 (104-143)	**14**	**0.002**	104 (92-110)	4	0.463	102 (96-113)	2	0.277	116 (106-148)	**16**	**0.000**	100 (84-115)	0	1.000	106 (96-111)	6	0.104	85 (78-102)	**-15**	0.563
Reference	100 (100-100)	0		100 (100-100)	0		100 (100-100)	0		100 (100-100)	0		100 (100-100)	0		100 (100-100)	0		100 (100-100)	0		100 (100-100)	0	
Storage at RT (4hr)	99 (95-103)	-1	0.903	95 (92-100)	-5	0.135	99 (94-104)	-1	0.720	103 (99-105)	3	0.121	98 (83-102)	-2	0.169	98 (91-108)	-2	0.677	96 (89-100)	-4	0.064	105 (97-106)	5	0.519
Storage at RT (7 hrs)	97 (94-101)	-3	0.194	98 (94-102)	-2	0.268	96 (92-103)	-4	0.229	101 (96-106)	1	0.421	93 (87-104)	-7	0.208	101 (93-131)	1	0.470	95 (93-103)	-5	0.169	93 (86-107)	-7	0.470
Storage at RT (24 hrs)	95 (92-102)	-5	0.153	101 (100-104)	1	0.296	95 (92-101)	-5	0.209	101 (97-107)	1	0.720	97 (79-103)	-3	0.389	106 (89-135)	6	0.519	98 (90-102)	-2	0.095	96 (86-114)	-4	0.791
Storage at RT (72 hrs)	92 (87-103)	-8	**0.042**	102 (98-105)	2	0.296	98 (92-102)	-2	0.258	106 (100-108)	6	0.083	103 (88-108)	3	0.890	101 (87-120)	1	0.520	94 (88-97)	-6	**0.007**	101 (86-107)	1	1.000
Reference	100 (100-100)	0		100 (100-100)	0		100 (100-100)	0		100 (100-100)	0		100 (100-100)	0		100 (100-100)	0		100 (100-100)	0		100 (100-100)	0	
storage at 4C 4 hrs	102 (96-104)	2	0.804	103 (98-106)	3	0.095	100 (93-102)	0	0.456	103 (101-106)	3	0.068	100 (97-108)	0	0.489	96 (90-107)	-4	0.820	98 (95-100)	-2	**0.020**	98 (88-109)	-2	0.910
storage at 4C 7 hrs	103 (97-106)	3	0.414	104 (102-108)	4	**0.015**	99 (96-105)	-1	0.934	101 (97-103)	1	0.670	101 (99-112)	1	0.208	107 (92-116)	7	0.313	100 (93-105)	0	0.635	106 (98-118)	6	0.250
storage at 4C 24 hrs	101 (93-103)	1	0.421	103 (97-105)	3	0.489	98 (91-102)	-2	0.252	102 (98-107)	2	0.463	103 (92-107)	3	0.804	90 (81-106)	**-10.4**	0.496	98 (95-103)	-2	0.296	94 (78-112)	-6	0.652
storage at 4C 72 hrs	100 (95-108)	0	0.660	110 (98-111)	10	0.208	97 (92-101)	-3	0.121	101 (99-104)	1	0.808	103 (95-110)	3	0.599	88 (58-96)	**-12**	0.098	94 (92-98)	-6	**0.020**	80 (60-103)	**-20**	0.203
Reference	100 (100-100)	0		100 (100-100)	0		100 (100-100)	0		100 (100-100)	0		100 (100-100)	0		100 (100-100)	0		100 (100-100)	0		100 (100-100)	0	
Temporary storage for 1 wk at RT	91 (79-103)	-9	0.103	102 (98-105)	2	0.169	94 (89-99)	-6	**0.002**	101 (95-106)	1	0.720	106 (101-113)	6	0.055	103 (75-121)	3	0.910	95 (88-98)	-5	**0.001**	100 (92-119)	0	0.820
Temporary storage for 2 wk at RT	101 (80-105)	1	0.303	99 (92-101)	-1	0.389	93 (89-97)	-7	**0.000**	94 (92-103)	-6	0.277	102 (96-111)	2	0.489	80 (78-85)	**-20**	0.129	83 (79-86)	**-17**	**0.000**	92 (69-107)	-8	0.250
Temporary storage for 1 wk at 2-8°C	102 (96-114)	2	0.379	100 (94-104)	0	0.890	99 (94-104)	-1	0.489	96 (89-103)	-4	0.252	118 (107-126)	**18**	**0.001**	87 (71-98)	**-13**	0.426	96 (74-103)	-4	0.208	93 (86-98)	-7	0.164
Temporary storage for 2 wk at 2-8°C	101 (98-109)	1	0.451	97 (89-105)	-3	0.421	100 (90-104)	0	0.890	91 (83-96)	-9	**0.015**	119 (103-143)	**19**	**0.001**	71 (52-89)	**-29**	0.129	97 (74-99)	-3	**0.030**	94 (86-109)	-6	0.652
Reference	100 (100-100)	0		100 (100-100)	0		100 (100-100)	0		100 (100-100)	0		100 (100-100)	0		100 (100-100)	0		100 (100-100)	0		100 (100-100)	0	
temporary storaget at -20°C (2wk)	98 (97-106)	-2	0.903	97 (93-103)	-3	0.497	101 (97-104)	1	0.952	100 (98-105)	0	0.839	99 (98-104)	-1	0.890	100 (96-114)	0	0.625	95 (93-99)	-5	0.083	106 (98-111)	6	0.204
temporary storaget at -20°C (2 mons)	110 (106-115)	9.6	**0.003**	99 (93-108)	-1	0.893	100 (98-105)	0	0.845	104 (99-107)	4	0.244	105 (97-112)	5	0.252	87 (78-95)	**-13**	0.064	94 (92-97)	-6	0.055	96 (82-105)	-4	0.380
temporary storaget at -20°C(6 mons)	110 (107-112)	9.6	**0.000**	99 (96-105)	-1	1.000	100 (96-104)	0	0.903	109 (102-111)	9	**0.006**	106 (102-113)	6	**0.001**	101 (88-119)	1	0.677	94 (90-101)	-6	**0.048**	109 (92-122)	9	0.266
Reference	100 (100-100)	0		100 (100-100)	0		100 (100-100)	0		100 (100-100)	0		100 (100-100)	0		100 (100-100)	0		100 (100-100)	0		100 (100-100)	0	
Tube transfer 1x	100 (97-101)	0	0.780	102 (99-105)	2	0.229	98 (97-109)	-2	0.855	105 (99-115)	5	0.375	101 (99-107)	1	0.296	107 (84-116)	7	0.844	101 (99-103)	1	0.168	102 (83-117)	2	0.945
Tube transfer 2x	101 (99-106)	1	0.358	105 (98-108)	5	0.169	102 (91-110)	2	0.808	108 (102-117)	8	**0.037**	102 (100-105)	2	0.091	98 (73-113)	-2	0.641	99 (97-104)	-1	0.635	102 (75-110)	2	0.844
Tube transfer 4x	100 (96-110)	0	0.903	101 (97-106)	1	0.524	101 (91-106)	1	0.952	99 (94-108)	-1	1.000	100 (98-106)	0	0.761	103 (84-118)	3	0.844	99 (95-103)	-1	0.635	104 (97-114)	4	0.461
Reference	100 (100-100)	0		100 (100-100)	0		100 (100-100)	0		100 (100-100)	0		100 (100-100)	0		100 (100-100)	0		100 (100-100)	0		100 (100-100)	0	
Centrifugation settings (10 min at 4°C)	110 (104-115)	**10.5**	**0.002**	100 (95-102)	0	0.463	105 (100-107)	5	**0.033**	101 (88-103)	1	0.855	100 (93-105)	0	1.000	98 (91-105)	-2	0.547	101 (99-106)	1	0.296	93 (83-104)	-7	0.313
Centrifugation settings (30 min at RT)	89 (82-94)	**-11**	**0.000**	107 (99-110)	7	0.073	99 (98-105)	-1	0.934	97 (93-100)	-3	0.091	101 (97-105)	1	0.679	91 (70-114)	-9	0.461	101 (96-103)	1	0.903	91 (69-118)	-9	0.461
Centrifugation settings (30 min at 4°C)	96 (91-102)	-4	0.151	103 (100-108)	3	0.073	100 (96-104)	0	1.000	101 (94-105)	1	0.903	103 (97-108)	3	0.359	84 (80-105)	**-16**	0.383	99 (91-102)	-1	0.241	93 (79-107)	-7	0.641
Reference	100 (100-100)	0		100 (100-100)	0		100 (100-100)	0		100 (100-100)	0		100 (100-100)	0		100 (100-100)	0		100 (100-100)	0		100 (100-100)	0	
collection tube (Serum)	83 (78-87)	**-17**	**0.001**	83 (76-86)	**-17**	**0.000**	106 (100-112)	6	**0.031**	70 (66-74)	**-30**	**0.001**	139 (125-205)	**39**	**0.001**	114 (114-114)	**14**	1.000	298 (290-307)	**198**	0.250	38 (38-38)	**-62**	1.000
collection tube (Na-Citrate)	80 (68-83)	**-20**	**0.000**	89 (83-101)	**-11**	0.277	82 (81-92)	**-18**	**0.000**	94 (88-102)	-6	**0.040**	85 (79-88)	**-15**	**0.000**	91 (83-96)	-9	0.297	92 (80-104)	-8	0.216	106 (80-122)	6	0.938
collection tube (Li-Heparin)	86 (82-88)	**-14**	**0.016**	114 (103-117)	**14**	**0.003**	109 (103-115)	9	0.068	114 (106-126)	**14**	**0.021**	96 (89-104)	-4	0.426	71 (61-80)	**-29**	0.219	117 (101-121)	**17**	**0.017**	61 (53-71)	**-39**	**0.031**
Reference	100 (100-100)	0		100 (100-100)	0		100 (100-100)	0		100 (100-100)	0		100 (100-100)	0		100 (100-100)	0		100 (100-100)	0		100 (100-100)	0	
FT (1X)	103 (98-105)	3	0.094	99 (94-101)	-1	0.169	98 (91-101)	-2	0.149	103 (97-110)	3	0.426	103 (96-111)	3	0.391	111 (100-119)	**11**	0.109	96 (91-104)	-4	0.216	117 (109-123)	**17**	**0.047**
FT (2X)	103 (100-107)	3	**0.038**	99 (96-102)	-1	0.454	89 (85-93)	**-11**	**0.012**	99 (94-105)	-1	1.000	101 (95-108)	1	0.502	93 (86-101)	-7	0.375	97 (90-104)	-3	0.542	97 (95-112)	-3	0.813
FT (4X)	105 (102-112)	5	0.125	93 (93-97)	-7	0.125	93 (74-99)	-7	0.178	102 (97-109)	2	0.191	107 (102-116)	7	0.313	108 (96-120)	8	1.000	102 (98-106)	2	0.813	111 (95-126)	**11**	1.000
	100 (100-100)	0		100 (100-100)	0		100 (100-100)	0		100 (100-100)	0		100 (100-100)	0		100 (100-100)	0		100 (100-100)	0		100 (100-100)	0	
hemolysis (Low)	111 (106-115)	**11**	**0.038**	102 (98-107)	2	0.454	100 (98-105)	0	**0.012**	100 (96-104)	0	0.855	100 (94-109)	0	0.502	96 (90-102)	-4	0.375	97 (91-103)	-3	0.542	98 (90-107)	-2	0.813
hemolysis (high)	128 (121-138)	**28**	**0.000**	107 (100-120)	7	**0.026**	102 (99-110)	2	0.117	95 (88-100)	-5	0.104	91 (85-104)	-9	0.389	101 (94-115)	1	0.641	99 (94-105)	-1	1.000	107 (88-112)	7	0.844
